# Heavy cannabis use and attentional avoidance of anxiety-related stimuli

**DOI:** 10.1016/j.abrep.2016.02.004

**Published:** 2016-03-02

**Authors:** T.D.W. Wilcockson, N.E.M. Sanal

**Affiliations:** a Department of Psychology, Lancaster University, Bailrigg, Lancaster LA1 4YW, UK; b Psychology Department, Swansea University, Singleton Park, Swansea SA2 8PP, UK

**Keywords:** Attentional bias, Anxiety, Cannabis

## Abstract

**Objectives:**

Cannabis is now the most widely used illicit substance in the world. Previous research demonstrates that cannabis use is associated with dysfunctional affect regulation and anxiety. Anxiety is characterised by attentional biases in the presence of emotional information. This novel study therefore examined the attentional bias of cannabis users when presented with anxiety-related stimuli. The aim was to establish whether cannabis users respond to anxiety-related stimuli differently to control participants.

**Methods:**

A dot-probe paradigm was utilised using undergraduate students. Trials contained anxiety-related stimuli and neutral control stimuli. Eye-tracking was used to measure attention for the stimuli.

**Results:**

Results indicated that cannabis users demonstrated attentional-avoidance behaviour when presented with anxiety-related stimuli.

**Conclusions:**

The findings suggest a difference in processing of emotional information in relation to neutral information between groups. It would appear that cannabis users avoid anxiety provoking stimuli. Such behaviour could potentially have motivational properties that could lead to exacerbating anxiety disorder-type behaviour.

## Highlights

• An anxiety-related visual dot-probe task with gaze tracking was used.
• The task was able to measure different types of attentional bias.
• Heavy cannabis use was associated with attentional avoidance of anxiety-related stimuli.

## 1 Heavy cannabis use and attentional avoidance of anxiety-related stimuli

When in threatening situations, individuals with anxiety disorders differ from others in terms of how they think (Butler & Matthews, 1983), remember (Amir, Foa, & Coles, 1998), and attend (MacLeod & Mathews, 1988). Research now suggests that these findings may be associated with biases in attentional processing (see Mogg & Bradley, 1998). Of tasks designed to measure attentional bias (AB), the most commonly used measure is a modified version of the Stroop task (Stroop, 1935). During this task a delay in reaction time (RT) for the emotional words would be expected, as long as the word meaning is relevant to participants. This interference has been suggested to be representative of an AB. This is because the delay is thought to be the result of the meaning of the word capturing the attention of the participant, thus reducing cognitive resources for the concurrent task (that of naming the colour). Anxiety-related AB is generally assumed to be the result of a negative appraisal of threat-related stimuli, as threat stimuli could potentially have inherent motivational properties (see Mogg & Bradley, 1998). Such findings have been replicated and extended by use of other tasks, such as the dot probe (see Cisler & Koster, 2010) and eye-tracking techniques (see Armstrong & Olatunji, 2012).

The AB associated with threat-related stimuli has been well documented (e.g. Bar-Haim, Lamy, Pergamin, Bakermans-Kranenburg, & Van Ijzendoorn, 2007). Those who are prone to anxiety problems have been found to have an increased AB for stimuli related to threat compared to those who are typically not anxious (e.g. Mogg & Bradley, 1998). For example, those with specific phobias have demonstrated an AB for stimuli related to their phobia (e.g. an AB for spiders). By contrast, those who have generalised anxiety disorders demonstrate an AB for stimuli that are generally threat-related (see Bar-Haim et al., 2007). Such AB has been found to be a robust phenomenon within populations high in anxiety (e.g. Cisler, Bacon, & Williams, 2009). Whether these ABs are *toward* the stimulus or *away* from the stimulus is an important issue.

Cisler and Koster (2010), through meta-analysis, made the suggestion that there are three forms of AB: facilitated attention, delayed disengagement, and attentional avoidance (attentional avoidance cannot occur concurrently with the other forms of AB). For threat stimuli, facilitated attention has been observed where attention has been found to be drawn to threat stimuli. This is a process of rapid orienting of attention. Further, threat stimuli are also associated with a delayed disengagement of attention. This is when attention has been captured by threat stimuli, which impairs the switching of attention. Attentional avoidance has been suggested to be the complete contrast of traditional notions of AB, as it is thought that threat stimuli, in some cases, actually cause attention to be diverted away from a threat cue (e.g. Koster, Crombez, Verschuere, & De Houwer, 2006). This entire process, though noting that each component can exist without the presence of the other two, has been thought to be the result of a hypersensitive system for coping under threat. We are rapidly able to locate threat and have trouble removing our attention from it. But, following threat, we remove our attention from the threatening stimulus, perhaps to alleviate anxiety (Cisler & Koster, 2010).

Although there is a lot of research which suggests the role of attention in the alleviation of anxiety (e.g. Eysenck, Derakshan, Santos, & Calvo, 2007), there are other, less covert, methods anxiety sufferers have utilised. One such method that anxiety sufferers have found to cope with their anxiety is substance abuse (see Nunes & Blanco, 2009). Indeed, data from the National Comorbidity Study (Kessler, Chiu, Demler, & Walters, 2005) indicates that individuals with anxiety disorders are 2–3 times more likely to have a substance use disorder at some time in their lives than the general population. These results suggest that there may be a comorbidity between substance use and anxiety disorders. However, the cause and effect would appear unclear. Substance use can be used as a maladaptive form of emotion regulation, as it can manage negative affect and enhance positive affect (Tschann et al., 1994). Theories supporting the negative reinforcement and self-medication theory claim that emotional processes with their related disturbances are the primary contributing factor of substance use, abuse, and dependence (Baker et al., 2004, Duncan, 1975, Khantzian and Treece, 1985). It is postulated that individuals engage in substance use behaviour to cope with stress and the preceding reason for initiation is emotional distress (Tschann et al., 1994).

There could potentially be three theoretical mechanisms in place that explain the link between substance use and anxiety. Firstly, substance use disorders could potentially develop in an attempt to self-medicate anxiety symptoms. Secondly, anxiety symptoms could occur whilst experiencing substance use withdrawal symptoms. Finally, there could be an interaction between the above mechanisms. Here depressants, such as alcohol, opiates and cannabis, could be used in an attempt to decrease anxiety, but during withdrawal states, anxiety could be increased which would lead to an exacerbation of the anxiety disorder and making relapse to substance use more likely.

Of substances of abuse, cannabis has long been associated with anxiety. Agosti, Nunes, and Levin (2002) found that cannabis dependence doubled the likelihood of an anxiety disorder. There is also evidence to suggest potentially a causal relationship (see Patton et al., 2002). However, whether this is a cause or effect is beyond the scope of this paper. Nevertheless, it would appear that there is evidence which could suggest that cannabis users use cannabis in order to alleviate the symptoms of anxiety, as frequent cannabis use has been found to be associated with self-reported statements related to physical discomfort, unpleasant emotions, and conflict with others (Johnston & O'Malley, 1986; cf. McKay, Murphy, McGuire, Rivinus, & Maisto, 1992). Kaplan, Johnson, and Bailey (1986) indicated that a link exists between avoidance coping strategies and increased cannabis use. Cannabis users, with elevated social anxiety, report to use cannabis to avoid social scrutiny and as a negative affect management strategy (Buckner, Bonn-Miller, Zvolensky, & Schmidt, 2007). Consistent with the motivational models of substance use it is also claimed that individuals with an elevated social anxiety also use cannabis to reduce anxiety in social situations (Baker et al., 2004).

Therefore, due to the association between cannabis and anxiety, it is particularly important to provide evidence which may increase our understanding toward how this association manifests. Previously research has indicated altered affective response and emotional evaluation in cannabis users (see Gruber et al., 2009, Wesley et al., 2015). Cannabis would appear to decrease reactivity to negative affective stimuli whilst also acutely inducing anxiety (see Powell et al., 2002, Salamone, 1994). This pattern of affective evaluation may be the result of deficits in implicit evaluation and attentional processes. Indeed, Metrik et al. (2015) observed that a sub-group of cannabis users with cannabis dependence demonstrated a within-subjects difference regarding the processing of emotional-related words on an emotional-Stroop task when under the influence of cannabis. It was suggested that cannabis may increase in the cognitive resources required for the processing of negatively valenced stimuli. However, these previous findings predominantly measure biases in cognitive processing, rather than biases in the orientation of attention. Tasks like the emotional-Stroop would appear to measure the delayed disengagement of attention caused by the meaning of a stimulus causing an increase in cognitive processing. In order to measure the emotionally aversive nature of a stimulus a visual probe task would be necessary with gaze tracking capabilities.

Within this paper, we aim to observe whether cannabis users demonstrate an AB for anxiety-related stimuli. Note, we do not suggest that cannabis is the only substance of abuse that may lead to differences in AB. Previous research would suggest that there are a number of types of AB which could be demonstrated by our sample (see Cisler & Koster, 2010). Therefore, we utilise a dot-probe (see MacLeod, Mathews, & Tata, 1986), as this is more sensitive at measuring the different AB than the emotional-Stroop (as within the Stroop there are not multiple stimuli in different locations competing for attention). In the dot-probe task, a trial involves two stimuli, typically presented on the left and right part of the distal layout, such that one stimulus is neutral, whilst the other is anxiety-related. The stimuli disappear and a dot appears either at the location of the neutral or the anxiety-related stimulus. The task of the participant is to identify the location of the dot as quickly as possible. Depending on whether the dot replaces the neutral or the anxiety-related stimulus, and the relative speed of responding across trials, the experimenter can establish the presence of an AB. The speed of which one responds on the dot-probe indicates which picture was being looked at when the probe appeared. Therefore, if, for example, the anxiety-related stimulus was being attended to when the probe appeared, one would anticipate a decreased reaction time (RT) for responding to the probe due to already attending to that side of the screen. However, as well as RT, we can also look at accuracy, and, with the use of an eye-tracker, first fixation and dwell time. First fixation time is the time to orient attention toward each picture and dwell time is the amount of time looking at each picture-type; either anxiety-related or neutral control. Previous research has suggested that anxiety-related stimuli can either lead to facilitated attention, delayed attentional disengagement, or attentional avoidance (see Cisler & Koster, 2010). We aim to measure whether cannabis users differ from controls in the way they process anxiety-related stimuli.

## 2 Methods

### 2.1 Participants

A total of 40 participants were recruited for the study. However, of those participants initially recruited 23 participants were included in the final analyses. The cannabis user group were asked about their cannabis use and required to have not taken cannabis within the previous 24 h but they were required to otherwise be daily cannabis users. Control non-users were required to have never taken cannabis to be eligible to participate. These criteria remove recreational cannabis users and are consistent with previous studies (see Gruber et al., 2009, Wesley et al., 2015). Therefore, nine participants were found to be ineligible to participate through this pre-selection phase, due to not matching these user criteria. A further eight participants' results were unavailable due to calibration errors with the eye-tracker. Therefore, the final sample consisted of eight heavy cannabis users (mean age: 23.13; sd: 4.16; male = 6) and 15 control non-users (mean age: 24.13; sd: 3.94; male = 10). Participants were recruited from Swansea University and received course credit for their time.

### 2.2 Materials and apparatus

AB was assessed using a dot-probe task (MacLeod et al., 1986). We also measured eye-movement during the task by using an EyeLink Desktop 1000 eye-tracker (SR Research Ltd., Ontario, Canada). Experimenter Builder (SR Research Ltd., Ontario, Canada) was used during the task to control the stimulus presentation events. In the dot-probe task, during each trial, for 1500 ms, a picture pair would be presented side-by-side on the screen. This presentation time was chosen as it was considered long enough in duration to potentially measure all three forms of AB (see Mogg & Bradley, 1998). A probe would then appear either on the left or right side of the screen for 275 ms. Participants respond using a control pad whether the probe was on the left or right side of the screen. We measured accuracy, RT, and with the use of the eye-tracker, we also measured first fixation time and dwell time for the anxiety-related and neutral control stimuli. First fixation time was the average time taken to orient to each picture-type. Whereas, dwell time was the average amount of time spent looking at each picture-type.

**Fig. 1:**
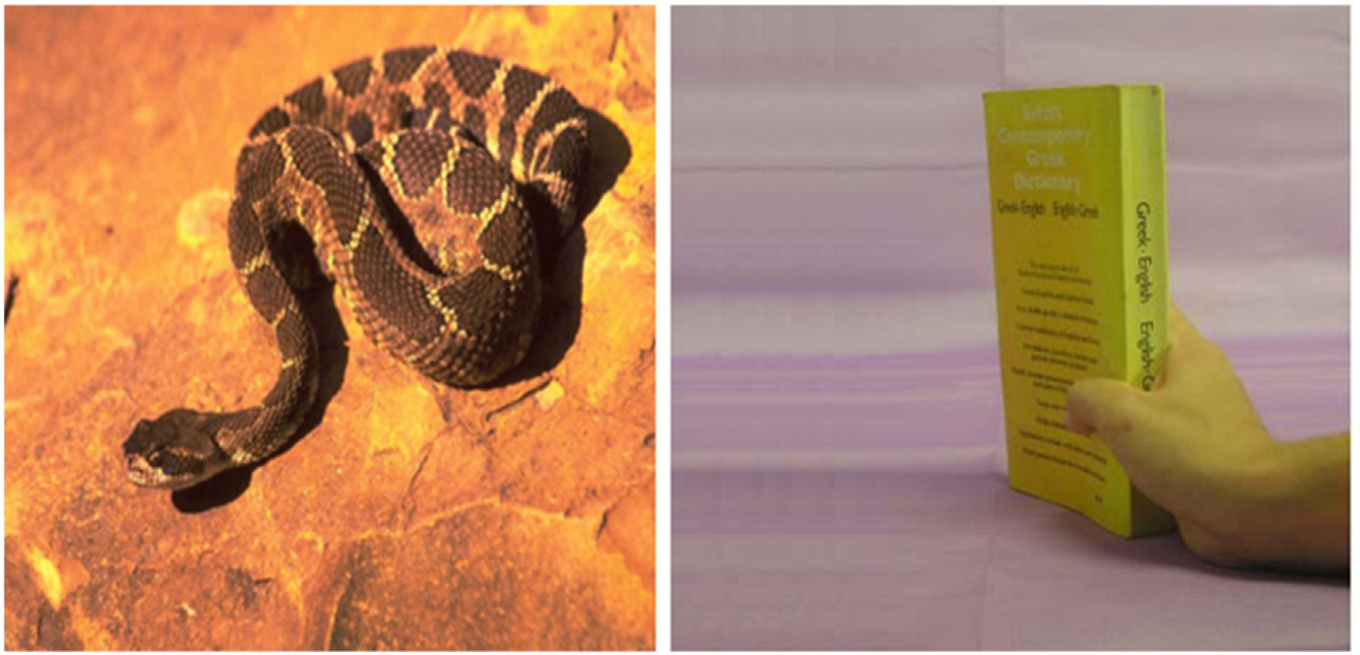
Example of anxiety-related (left) and neutral, control (right) stimuli used within the dot-probe task.

Of the picture pairs; one was anxiety-related and one neutral-control. Which side of the screen the anxiety and neutral control stimuli appeared was counterbalanced. There were 20 trials during the task. Therefore, there were 20 anxiety-related images and 20 neutral control images. Anxiety-related images were selected from a database of affective stimuli (AMP; Payne, Cheng, Govorun, & Stewart, 2005) and included pictures with snakes, spiders, war, wounds, explosions, etc. (see Fig. 1). These were matched in terms of colour, brightness, contrast, and object/background size ratio with an item of neutral-control stimuli from Wilcockson and Pothos (2015) and included items related to office equipment (see Fig. 1). All 40 pictures measured 105 mm × 105 mm. Each picture was presented once in order to prevent (de)sensitisation and practice effects.

Anxiety was assessed using the Generalized Anxiety Disorder scale (GAD-7 scale; Spitzer, Kroenke, Williams, & Löwe, 2006). The scale consisted of seven anxiety-related statements to which participants had to indicate how strongly they agreed (e.g. ‘over the past two weeks have you had trouble relaxing?’) on a 4 point response scale ranging from ‘not difficult at all’ to ‘extremely difficult’. Participants could score between 0 and 3 on each question. Participants scoring over 15 out of 21 on this scale would have been removed, as this is indicative of severe anxiety. However, no participants were removed due to this. This measure was used in order to control for any premorbid anxiety conditions. Previous research has found that the GAD-7 is an appropriate measure for measuring anxiety in non-clinical populations (see Löwe et al., 2008).

Drug use was assessed using the UEL (University of East London) Drug History Questionnaire (Parrott, Sisk, & Turner, 2000). This questionnaire consisted of a list of illicit substances with dichotomous answers YES/NO, indicative of use for that substance. Substance use prevalence was assessed numerically for the last month and life–time period for all substances except for tobacco, cannabis and alcohol use. This questionnaire was used as part of the pre-selection phase only.

### 2.3 Procedure

Upon arrival to the laboratory, participants completed the UEL Drug History Questionnaire and the GAD-7 scale. Participants then performed the dot-probe. This task was completed with a control pad, whilst eye movements were recorded.

### 2.4 Analysis

Of the dependent variables; RT was computed from the amount of time taken to respond on each trial whilst accounting for stimuli-type. Therefore the RT variable was: Bias = probe behind neutral RT − probe behind threat RT. Accuracy was the amount of errors made on the trials. First fixation time was the average time taken to orient to each stimuli-type. Whereas, dwell time was the average amount of time spent looking at each stimuli-type. Note, for the four eye-tracking variables (anxiety and neutral-control; dwell time and first fixation time) the average time for each stimuli-type across the corresponding 20 trials were computed.

With these variables it was possible to measure facilitated attention and attentional avoidance. For example, attentional avoidance is inferred if increased mean first fixation time for the anxiety stimulus is greater than that for the neutral stimulus. Moreover, if increased mean first fixation for the anxiety stimulus is greater for the cannabis users than for the controls, it is inferred that group displays increased, e.g., attentional avoidance.

Analysis will involve independent t-tests in order to observe differences between groups for both the anxiety stimuli and the neutral stimuli. Further, a paired-samples t-test will be used to measure differences between stimuli-types within groups.

## 3 Results

Firstly, the GAD-7 was considered. Cannabis users and controls did not differ in their responses on this variable (t(21) = 1.412;p = .173;*d* = .62). Therefore there were no explicit differences in reported anxiety. The dot-probe attentional bias variables were then analysed in order to establish whether there were any differences in AB within the participant groups; considered first are the RT and error variables before the eye-tracking variables (see Table 1). Of the eye tracking variables, first fixation time may indicate the initial orienting of attention, whereas the dwell time variable indicates attentional capture and attentional avoidance.

**Table 1:** Cannabis user and controls means, ranges, and standard deviations for the anxiety measure (GAD-7), the dot-probe attentional bias measures, and the dot-probe attentional bias eye tracking variables.

	Cannabis user			Control participant		
	Range	Mean	SD	Range	Mean	SD
GAD-7	6.00	3.88	2.47	12.00	5.57	3.59
Dot-probe attentional bias errors	2.00	0.25	0.71	1.00	0.13	0.35
Dot-probe attentional bias RT (seconds)	1.33	0.19	0.45	4.38	0.37	1.13
Neutral stimuli dwell time	0.53	0.67	0.21	0.57	0.79	0.18
Anxiety-related dwell time	0.46	0.52	0.17	0.53	0.67	0.14
Neutral stimuli first fixation time	0.83	1.20	0.26	1.28	1.31	0.29
Anxiety-related first fixation time	0.68	1.56	0.26	1.02	1.40	0.29

We then perform an independent-sample t-test to determine if control participants and cannabis users were different on any of the measures. There was no significant difference between cannabis users and control participants in terms of the dot-probe attentional bias errors (t(21) = 1.00;p = .329;*d* = .44), nor dot-probe attentional bias RT (t(21) = −.438;p = .666;*d* = .19).

For the eye-tracking variable of first fixation time, neutral-control stimuli led to no significant difference between controls (m = 1.31;sd = .293) and cannabis users (m = 1.20;sd = .264), t(21) = .861;p = .399;*d* = .38). Nor was there a significant difference, for the anxiety-related, between controls (m = 1.40;sd = .287) and cannabis users (m = 1.60;sd = .263), (t(21) = − 1.308;p = .205;*d* = .57). These results indicate that there was no difference between the groups in terms of initial orienting of attention.

For the eye-tracking variable of dwell time, neutral-control stimuli led to no significant difference between controls (m = .791;sd = .184) and cannabis users (m = .670;sd = .213), (t(21) = 1.424;p = .169;*d* = .62). Whereas, for the anxiety-related stimuli, controls (m = .669;sd = .142) differed from cannabis users (m = .519;sd = .170) significantly, (t(21) = 2.244;p = .036;*d* = .98). These results suggest that cannabis users had a decreased dwell time as compared to the controls in terms of their attention toward anxiety-related stimuli. Further, a paired sample t-test of neutral-control vs. anxiety-related stimuli for the cannabis users confirms this difference in attention for anxiety-related stimuli (m = 5.19;sd = 1.70) and neutral-control stimuli (m = 6.70;sd = 2.13), (t(7) = − 3.132;p = .017;*d* = .78). The results may therefore indicate that cannabis users demonstrate attentional avoidance for anxiety-related stimuli.

## 4 Discussion

During this experiment we performed an anxiety-related dot-probe task in order to investigate whether cannabis users differed from control participants with regard to their attention toward anxiety-related cues. We found a significant difference between cannabis users and controls in terms of dwell time for anxiety-related stimuli with cannabis users demonstrating a decreased dwell time. However, this result was not observed for the other dot-probe dependent variables. Nevertheless, we suggest that this experiment demonstrates that the cannabis users in our sample demonstrated increased attentional avoidance for anxiety-related stimuli.

To our knowledge, this is the first analysis of the effect of cannabis upon anxiety-related AB as measured with a gaze tracking visual probe task. Nevertheless, previous research has suggested that the most consistent finding in the AB and anxiety literature relates to avoidance (e.g. Hermans, Vansteenwegen, & Eelen, 1999). In these studies, avoidance was also suggested as a decrease in sustained attention for a threat stimulus, relative to a neutral stimulus (e.g. Rinck & Becker, 2006). This therefore supports our findings, as avoidance would appear to be a robust measure of anxiety-related AB. Therefore, if cannabis users are also demonstrating similar patterns in attention, they may have similar responses to anxiety. This may be because they already have anxiety-related deficits, or are in danger of developing them. Mogg and Bradley (1998) state that attentional avoidance may actually maintain anxiety disorders by preventing thorough processing of anxiety-related stimuli. This could potentially prevent reappraisal of the feared object and may maintain any negative learned associations. Therefore, cannabis users may also be engaging in attentional practices, which may actually lead to anxiety disorders, if they do not already have one.

The anxiety measure was not found to lead to significant differences. This may suggest that either, cannabis users and controls do not differ on this measure, or, that cannabis users use cannabis to self-medicate due to an underlying anxious state. However, this is pure speculation and more research would be needed to support this as we cannot ascertain this from the current study. From previous research it would seem that there is evidence to suggest that self-medication with substance use is a possibility (see Nunes & Blanco, 2009). However, the heavy cannabis users were abstinent for 24 h, which could be sufficient time to allow any underlying anxiety disorder to surface as measured by the GAD-7. It may be that whilst these cannabis users do not have any anxiety issues as shown by the GAD-7, they are exhibiting possible signs of a prodromal phase of anxiety (as indicated by their dwell times). The fact that after abstaining for 24 h the cannabis users are normal with regard to the GAD-7 suggests that cannabis use could possibly be leading to an anxiety disorder. However, this is purely speculation.

Clearly, the strength of the conclusions in this work is limited by the small sample size. The strict eligibility criteria meant that some participants were excluded. However, this criterion was chosen to screen out recreational cannabis users and to yield demographics consistent with previous studies (see Gruber et al., 2009, Wesley et al., 2015). This together with the sample consisting entirely of undergraduate students may have biased the results (note, though, that the study of this population does have important practical value). Also, demographic information is limited. For example, the cannabis use history of study participants is unknown. Nevertheless, we state certain interesting trends, with the intention of highlighting these as directions for future research. Future research would require a larger sample size and a more diverse sample of participants. This research would be required to fully support the conclusions which are indicated within this paper. However, significant differences in relation to the behavioural distinctions of interest were identified even with these sampling issues.

It should be noted that not all of the dot-probe dependent variables were significant in this study. However, it may be that the errors and RT measures were not sensitive to anxiety with the limited number of trials within this experiment and the eye-tracking measures may be more accurate. The discrepancy found between the two eye-tracking variables may be a result of the two measuring distinct aspects of AB. Initial orienting has been previously found within the anxiety literature (e.g. Mogg & Bradley, 1998), however, it may not have been observed here due to cannabis users alleviating the majority of the anxiety ‘symptoms’ with self-medication. Also, previous research (see Cisler & Koster, 2010) has indicated that attentional avoidance cannot occur alongside the other types of AB, due to the nature of the attentional processes involved i.e. the difference between increased attention and decreased attention. We, in this study, have found a decrease in attention for anxiety-related stimuli in cannabis users, which suggests that they attentionally avoid anxiety-related stimuli. We suggest that this may be the result of an anxiety-related coping mechanism.

This paper is not suggesting that all cannabis users have anxiety disorders. It may be that heavy cannabis use may result in attentional patterns which are indeed just similar to that of anxiety disorders. However, as attentional avoidance may have the potential to maintain anxiety disorders, if cannabis users start adopting this AB-type, then they may be under increased risk of developing anxiety problems. Further research is clearly needed here. However, it would appear that cannabis use affects anxiety-related AB.

## Source of funding

None declared.

## Contributors

Authors TW and NS designed the study and wrote the protocol. Authors TW and NS conducted literature searches. Author NS collected the data. Author TW conducted the statistical analysis. Author NS wrote the first draft of the manuscript and author TW wrote the final manuscript.

## Conflicts of interest

The authors declare no conflicts of interest.
